# Ultrasonic-Based Environmental Perception for Mobile 5G-Oriented XR Applications

**DOI:** 10.3390/s21041329

**Published:** 2021-02-13

**Authors:** Luca Davoli, Ioannis Paraskevopoulos, Cinzia Campanella, Stefano Bauro, Tommaso Vio, Andrea Abrardo, Gianluigi Ferrari

**Affiliations:** 1 Research Unit of Parma, Consorzio Nazionale Interuniversitario per le Telecomunicazioni (CNIT), 43124 Parma, Italy; gianluigi.ferrari@unipr.it; 2 Internet of Things (IoT) Laboratory, Department of Engineering and Architecture, University of Parma, 43124 Parma, Italy; 3 IGOODI s.r.l., 20123 Milano, Italy; ioannis.p@igoodi.it; 4 Vodafone Italia S.p.A., 20147 Milano, Italy; cinzia.campanella@vodafone.com (C.C.); stefano.bauro@vodafone.com (S.B.); tommaso.vio@vodafone.com (T.V.); 5 Research Unit of Siena, Consorzio Nazionale Interuniversitario per le Telecomunicazioni (CNIT), 53100 Siena, Italy; abrardo@diism.unisi.it; 6 Department of Information Engineering and Mathematics, University of Siena, 53100 Siena, Italy

**Keywords:** 5G, ultrasonic sensors, Internet of Things (IoT), eXtended Reality (XR), integration, future-proof applications, Tourism 4.0

## Abstract

One of the sectors that is expected to significantly benefit from 5G network deployment is eXtended Reality (XR). Besides the very high bandwidth, reliability, and Quality of Service (QoS) to be delivered to end users, XR also requires accurate environmental perception for safety reasons: this is fundamental when a user, wearing XR equipment, is immersed in a “virtual” world, but moves in a “real” environment. To overcome this limitation (especially when using low-cost XR equipments, such as cardboards worn by the end user), it is possible to exploit the potentialities offered by Internet of Things (IoT) nodes with sensing/actuating capabilities. In this paper, we rely on ultrasonic sensor-based IoT systems to perceive the surrounding environment and to provide “side information” to XR systems, then performing a preliminary experimental characterization campaign with different ultrasonic IoT system configurations worn by the end user. The combination of the information flows associated with XR and IoT components is enabled by 5G technology. An illustrative experimental scenario, relative to a “Tourism 4.0” IoT-aided VR application deployed by Vodafone in Milan, Italy, is presented.

## 1. Introduction

Due to the growing expectations of an increasing number of connected users (both humans and things), currently deployed cellular network architectures, especially 4G (LTE)/4G+ (LTE-A), are facing unprecedented challenges. Moreover, these cellular technologies have inherent limitations, e.g., in terms of localization and environmental perception capabilities. To this end, 5G technology, which is characterized by tremendous improvements in terms of high speed connectivity, ultra-low latency, resource management, and different access technologies, represents an effective answer to the expected high traffic increase [1]. 5G cellular technology features the following characteristics: (i) large-scale increased network capacity, to support bandwidth-intensive services (e.g., large amount of video and multimedia streams); (ii) improved flexibility, to obtain a better content delivery Quality of Service (QoS); and (iii) computing resources provision’s reliability, to support more valuable and trustworthy services. Therefore, 5G is envisioned to enable diverse applications and services with extremely heterogeneous performance and data consumption requirements, with ambitious technological goals (e.g., to support massive Machine-to-Machine, M2M, communication, mission-critical communications, and ultra-high speed mobile connectivity) [2]. Moreover, 5G will feature localization-aided mechanisms, with some solutions already defined within the 3GPP R16 [3] standardization activity, while higher accuracy (horizontal and vertical) solutions, typical of IoT use cases, will be defined in the next releases of 5G New Radio (NR) [4,5].

One of the sectors that is expected to significantly benefit from 5G network deployment is eXtended Reality (XR) [6], encompassing Augmented (AR), Virtual (VR), and Mixed Reality (MR), and a broad variety of transversal application domains and scenarios. More precisely, this is motivated by the well-known needs of XR, such as low latency, very large bandwidth, very high reliability and data recovery, very high Quality of Experience (QoE) for end users, and accurate localization capabilities, e.g., environmental navigation and reconstruction through localization data and virtual models’ retrieval from external Content Delivery Networks (CDNs) [7]. To this end, a well-known problem of XR applications occurs when a user wearing XR equipment (especially a VR one) is immersed in a “virtual” world, but moves in a “real” environment. Therefore, XR-based applications typically require that the user is not mobile, thus limiting significantly the User eXperience (UX).

The concept of Internet of Things (IoT) has become popular in the modern technological era. In recent years, many business models have been radically changed and, in some cases, have even been sparked by the IoT. Illustrative examples include Smart Cities [8], Smart Agriculture [9,10,11,12], Industry 4.0, and Industrial IoT (IIoT) [13,14]. Nowadays, thanks to the IoT, the real world is potentially a smart and hyperconnected world. For instance, it can be claimed that almost any productive sector may rely on the use of sensing nodes and may need to transfer data to external outsourced platforms (e.g., the Cloud), fulfilling several constraints (e.g., low latency, reliability, and knowledge sharing) [15]. Therefore, IoT is continuously evolving, relying on the use of physical sensing/actuating/communicating objects with virtual representations and connected services [16].

The goal of this paper is to propose an approach to allow physical mobility (in the real world) of XR-equipped users (immersed, fully or partially, in a virtual world). To this end, IoT systems can be used to perceive the environment, providing “side information” to XR systems, in order to allow a user immersed in a virtual world to move safely in the real world. In detail, environmental perception could be performed using sensors able to detect and estimate the distance of a moving person towards an obstacle. A relevant example is given by ultrasonic sensors, based on the physical principle associated with the round-trip propagation time (from sensor to obstacle and back) of an ultrasound beam, from which the sensor–obstacle distance is estimated. Moreover, it is interesting to investigate and understand how these sensors can be used, considering different configurations worn by the end user, to perceive the surrounding environment.

The combination of IoT (in this case, ultrasonic sensor-based) information and XR information flows, in order to guarantee a high quality UX as required by XR applications, is enabled by 5G technology. In other words, we want to highlight how the combination of 5G, XR, and IoT technologies paves the way to “future-proof” applications [17]. In particular, we discuss on the benefits and drawbacks of this technological overlap in the context of the “6 Degree-of-Freedom” (6DoF) concept which, by allowing end users to be immersed in a three-dimensional virtual world while moving in a real environment, has strict constraints (e.g., accurate positioning of the user in the scene, ultra-low latency, and high data rate) that can be satisfied only through the aforementioned joint integration. This 6DoF concept is applied to an illustrative scenario denoted as “Tourism 4.0” and deployed by Vodafone in Milan, Italy. This is a representative scenario, which is nowadays particularly relevant as tourism is one of the sectors mainly affected by COVID-19 [18,19]. In this context, one needs to guarantee the safety of people immersed in a cultural VR, without limiting significantly their experience in terms of contents’ enjoyment.

The rest of the paper is organized as follows. Section 2 is dedicated to related works. In Section 3, an overview of ultrasonic sensors is carried out, while Section 4 and Section 5 are dedicated to ultrasonic-based environmental and obstacle perception, respectively. In Section 6, the ultrasonic-based environmental perception for mobile 5G-oriented XR applications is presented, thus providing a latency analysis of the proposed architecture. In Section 7, the Tourism 4.0 application deployed in Milan, Italy, is discussed. Finally, in Section 8 a few additional scenarios are mentioned and conclusions are drawn.

## 2. Related Works and Motivations

The use of IoT systems with XR-related applications aims at allowing an end user to safely move in the real world by receiving context information [20,21]. Nevertheless, it is well known that existing IoT/VR solutions face critical challenges, related to connectivity, security, trustworthiness, latency, throughput, and ultra reliability. In this context, an Edge Computing infrastructure may help in solving these problems [22]. Moreover, a 5G/XR/IoT integration requires carefully defining the adopted networking paradigms, especially regarding the topological distribution of the different components in charge of handling and processing data incoming from low layers (e.g., in gaming scenarios, decentralized architectures may provide better performance and reliability to end users [23] than centralized ones) and in terms of tasks to be typically executed in an Edge Computinglike way (e.g., through Network Function Virtualization (NFV) [24,25]).

The integration of IoT and 5G is also expected to provide significant benefits in industrial scenarios [26,27], as IIoT needs to abide by strict communication requirements (e.g., high reliability, low latency, flexibility [28], and security [29,30]) which are fulfilled by 5G technology [13,31].

Considering the joint adoption of 5G technology with XR systems, in [32] the authors discuss on AR devices, their applications, and several existing techniques for overcoming energy consumption and latency problems, proposing a multi-layer architecture based on well-defined 5G reference components, aiming at offloading part of the XR tasks to the network edge, thus following an Edge Computing-oriented approach. A similar paradigm is proposed in [33], where multiple Edge Computing-like components cooperate in order to complete AR tasks and minimize the overall service failure (in terms of reliability and latency) probability of the architecture. In the area of entertainment, technological giants such as Google (Stadia [34]), Microsoft (xCloud [35]), and Apple (Arcade [36]) have recently announced the availability of relevant gaming platforms with remote rendering for ubiquitous access and with any kind of end device, demonstrating that 5G will be the enabling arbiter to define the future of XR.

Moreover, it is clear that latency represents a key requirement in VR-based applications. For this reason, latency has to be carefully considered in order to avoid VR user’s discomfort and motion-related limitations. Moreover, as VR-based 6DoF applications require highly performing computing architectures (e.g., for processing and rendering purposes), it is desirable to rely on processing infrastructures based on Edge Computing approaches, thus maintaining a low end-to-end transmission latency and processing delay. Therefore, one can leverage techniques targeting the 5G Multi-access Edge Computing (MEC) technology, standardized by the European Telecommunications Standards Institute (ETSI) [37,38] and exploiting the utilization of virtualization infrastructures and computing resources at the edge of the Internet. This leaves the used resources’ ownership to the infrastructure providers, while allowing their tenants to use these resources [39], even installing their own applications and processing their own data independently.

With particular regard to the network latency, in [40] the authors discuss on how emerging network technologies, and in particular 5G, will achieve low latencies considering three different aspects, such as Radio Access Network (RAN), core network, and caching. In [41], the impact that 5G cellular network elements, namely, Software-Defined Networking (SDN), NFV, and MEC, have in abiding by latency constraints and other 5G requirements, is investigated. In detail, an overview on different services based on 5G technology, together with the way 5G will boost the expansion and the interconnection of heterogeneous elements, even targeting IoT-oriented solutions, is presented. Examples of latency-critical services that need to be supported by 5G are shown in Table 1 and can be summarized as follows: (i) factory automation, with an end-to-end latency requirement between 0.25 ms and 10 ms; (ii) Intelligent Transportation Systems (ITSs), with an end-to-end latency between 10 ms and 100 ms; (iii) robotics and tele-presence, with a system response time shorter than a few milliseconds; (iv) Virtual Reality (VR), with a round-trip latency of around 1 ms; (v) Augmented Reality (AR), with a latency as low as a few milliseconds; (vi) health care (i.e., tele-diagnosis, tele-surgery, and tele-rehabilitation), with a round-trip latency between 1 ms and 10 ms; (vii) serious gaming, with an ideal Round-Trip Time (RTT) on the order of 1 ms (as a network latency of more than 30–50 ms would result in a significant degrade in UX); (viii) smart grid, with an end-to-end-delay of no more than 20 ms; and (ix) education and culture, with a round trip latency of 5–10 ms allowed for effective visual, auditory, and haptic interaction.

In a similar way, in [42] 5G use cases requiring ultra-low latency are analyzed, even from business perspectives. This represents an interesting point of view, as operators know how their businesses are performing with nowadays cellular networks (e.g., LTE), but need to invest in costly changes in their network, in order to let their businesses transit to 5G-oriented scenarios. The authors conclude that operators will obtain clear opportunities to add value and position themselves strongly in the market with the increasing number of services to be provided by 5G.

In [43], an overview of tactile Internet services and haptic interactions and communication is presented, highlighting the benefits of end-to-end service provisioning via network orchestration, and the need to realize these services in an automated fashion, to enable flexible, scalable, and cost-efficient service deployments. Moreover, the functionality of both 5G New Radio (NR) and LTE radio interfaces to provide Ultra-Reliable Low-Latency Communication (URLLC) services is described in [44], highlighting that a certain spectral efficiency can be demonstrated, at the cost, however, of a reduced efficiency if compared to mobile broadband services without latency or reliability constraints. In detail, the authors discuss on the fact that, in order to increase the reliability of URLLC services, robust coding/modulation/diversity schemes can be applied in accordance with the LTE and NR designs, thus providing redundancy over different carriers and transmission points by means of multi-connectivity and carrier aggregation frameworks specified for LTE. Furthermore, the importance of URLLC, in accordance with technology and industry requirements, is highlighted in [45], where concerns for IoT devices depending on the low latency and reliable communications of URLLC are also addressed. In [46], the authors provide a perspective on various trade-offs between energy efficiency and user plane delay that should be considered in 5G architectures, thus discussing on the benefits provided by the deployment of caching mechanisms preferably close to the network edge.

Looking at the aforementioned works, it is clear how the integration of heterogeneous data—such as XR information flows combined with IoT environmental information (i.e., generated by ultrasonic sensors)—and the latencies and delays introduced by the different components in the system need to be carefully taken into account. Moreover, in order to enable XR-oriented applications, thus guaranteeing, at the same time, high-quality UX and high safety for end users, the 5G technology plays a key role because, as highlighted before, its features and characteristics make it compatible with URLLC-oriented applications and scenarios. In particular, the adoption of this jointly technological ecosystem can enable 6DoF-oriented applications, allowing end users to be immersed in a three-dimensional virtual world while moving in a real environment, maintaining a round trip latency below an acceptable threshold (namely, 50 ms).

## 3. Ultrasonic Sensors

According to the International Electrotechnical Commission (IEC), a sensor is “*an element of a measurement chain which converts the physical input variable into an electric signal compatible with electronic circuits*” [47]. Nowadays, sensors are increasingly used in several technological sectors, allowing one to measure, identify, connect, locate, and activate processes with a high information rate in any operational environment. Furthermore, their diffusion has been facilitated by the advent of Micro Electromechanical Systems (MEMS) [48], enabling devices to be manufactured using silicon micro-manufactured products with a small number of components of various kinds that—due to their small sizes—can be integrated into highly miniaturized forms on a single substrate (typically silicon). In addition to their small dimensions, they have many more advantages, such as lightweight, low power consumption, remarkable speed, reliability, and ease of use in harsh environments.

Among the possible sensors that can be identified based on their operating principles—such as mechanical, electrical, and optical sensors—there is a particular category which, in recent years, is being increasingly used in various fields (including industry, automotive, warehousing, security, robotics, transport, and many more): *proximity sensors*. A proximity sensor can detect the presence of an object in the surrounding environment without any “tactile” contact, thus providing continuously a distance estimate between sensor and target object. Some of the most representative features of proximity sensors are the following:

- high reliability and durability, due to (i) lack of contact and absence of moving parts and (ii) extremely small size, being based on MEMS technologies;
- reduced response time and possibility to be used within a wide temperature range (in some cases, operating temperature can range between −40 ${}^{\circ }C$ and 200 ${}^{\circ }C$);
- adoption in “hostile” environments (e.g., in the presence of dust, oil, water, and, in some cases, chemical agents); and
- absence of abrasion or damage on the target object, as the detection occurs without any physical contact.

Moreover, proximity sensors can be usually grouped, according to the physical principle used to perform presence estimation [49], as follows.

- *Capacitive* proximity sensors, based on the detection of the electrical capacitance variation of a capacitor, leading to the presence of an object in the immediate vicinity of the sensor [50].
- *Inductive* proximity sensors, based on the detection of the interruption of the magnetic field, generated by an appropriate circuit including an oscillator and a coil, caused by the proximity of a ferromagnetic metal object in the area where the field is generated [51].
- *Magnetic* proximity sensors, detecting the magnetic field generated by a permanent magnet specially mounted on the object to be detected [52].
- *Optical* proximity sensors, also denoted as *photoelectric* and using a light beam to detect objects. Usually, the beam is infrared, as it is more robust against disturbances generated by environmental light sources [53].

In addition to the aforementioned proximity sensors, there is another interesting category which has considerable advantages in various fields and whose utilization is constantly increasing: *ultrasonic* proximity sensors. In detail, ultrasounds are mechanical sound waves with frequencies exceeding 20 kHz—thus higher than those audible by the human ear, but audible for various animal species (e.g., dogs, cats, bats, dolphins, etc.), which are mostly used, for example, in medical and industrial fields, where devices emitting high frequency ultrasonic pulses allow to detect the presence of objects in the immediate vicinity of the emitting station. [54]. From a physical point of view, the ultrasound’s generation starts from a membrane that vibrates when electrical energy is applied to it, thus acting as a diaphragm in the speakers: the vibrations compress and expand air molecules in the form of waves, moving back and forth. Then, the air in front of the membrane does not actually travel away from it, but there is still energy transport through the air.

On a technological level, ultrasonic sensors generally consist of (i) an ultrasonic capsule, (ii) an excitation circuit, (iii) a sensing circuit, and (iv) an output circuit. Moreover, there are the three main operating modes:

- *Direct diffusion*, in which the ultrasonic capsule is excited by high-voltage pulses until an ultrasonic signal begins to be emitted. Then, the ultrasonic beam is reflected back from the target to the sensor. Finally, the sensing circuit measures the time interval between the instant at which the ultrasonic beam was emitted and the instant at which it is received, allowing not only to detect the presence of the object, but also to measure the distance of the object from the sensor. In this case, the ultrasonic capsule *first* acts as an ultrasonic beam generator and, *then*, as a receiver.
- *Retro-reflection*, in which the system requires the presence of a background to operate, consisting of any flat fixed part orthogonal to the ultrasonic beam. The detector measures the time it takes for the ultrasonic signal to be received by the sensor, once reflected by the background. Any variation in this estimate indicates that an object has appeared between the sensor and the background, and therefore an obstacle has been detected.
- *Projector and receiver*, with the system consisting of two independent parts, an emitter and a receiver: the receiver collects the ultrasonic beam emitted by the emitter and, in the absence of any reception, an object is declared to be present in the operational range.

Among these operating modes, from now on the focus will be on direct diffusion sensors, as both projector and receiver are integrated in the same ultrasonic capsule and do not require background mapping. Moreover, if compared to inductive and capacitive sensors, ultrasonic sensors (i) allow the detection of objects placed even at considerable distances (e.g., tens of meters) and (ii) have a good level of immunity to electromagnetic interference. If compared to optical sensors, ultrasonic sensors detect objects using sound instead of light, and are suitable for applications where photoelectric sensors could not work—such as detecting transparent objects or objects with highly reflective or metallic surfaces, as well as in wet environments, where an optical beam could be reflected by water droplets for measuring the level of liquids. These are all applications where photoelectric sensors experience difficulties due to the translucency of the target, while ultrasonic sensors are not excessively affected by the color or reflectivity of the target.

The distance at which an ultrasonic sensor can detect a target depends on the following factors [55].

- Target size: the larger the target, the more the ultrasonic beam it reflects, the longer the range it can reach.
- Type of material to be detected: if the target is compact (e.g., wood, glass, metal, liquids, etc.), a large part of the signal is reflected; if the target is not very compact or is sound-absorbent (e.g., dust, polystyrene, or foam), then part of the signal energy is absorbed by the material, so that the effective target acquiring distance can be reduced.
- Object shape: if the object is flat and perpendicular to the direction of the ultrasonic beam, the signal is reflected back to the sensor and, therefore, the object can be detected correctly; by contrast, if the object is irregularly shaped or inclined, part of the signal can be scattered to the point where the sensor no longer receives enough signal to detect the object itself.
- Object temperature: temperature fluctuations affect the speed at which sound waves of an ultrasonic sensor travel.
- Presence of air currents in the surrounding environment: as an example, the wind, which can be caused by pneumatic equipment or fans, can deflect or disturb the path of sound waves, thus leading to the sensor not perfectly recognizing the target.

### 3.1. Ultrasonic-Based Positioning and Mapping

In the following, the use of ultrasonic technology for positioning purposes is explained, discussing how ultrasonic sensors can be used for obstacle detection [56]. In detail, their adoption for this kind of positioning task is motivated by the fact that ultrasonic sensor-based positioning systems are recognized as fine-grained systems, able to reach centimeter-level location accuracy, and offering a number of advantages over other technologies, such as low system cost, high reliability, high scalability, and high energy-efficiency. As mentioned in Section 3, ultrasonic sensors are affected by environmental conditions, such as temperature changes and humidity conditions, which can cause signal to fade away quickly and travel short distances.

Moreover, it is noteworthy to highlight that ultrasonic systems could be developed using either *narrowband waves* or *wideband waves*. The *first* type of wave can travel longer distances and needs lower emitter power and hardware complexity, while drawbacks appear at the multiple access layer, as the receiver cannot distinguish between signals from different emitters. On the other hand, the *second* type of wave overcomes certain limitations of narrowband systems, thus offering multiple access using spread spectrum signaling techniques, and providing robustness against interference. In the latter case, some limitations may be associated with the rapid attenuation of wideband signals, thus resulting in higher power usage and complex hardware.

**Figure 1:**
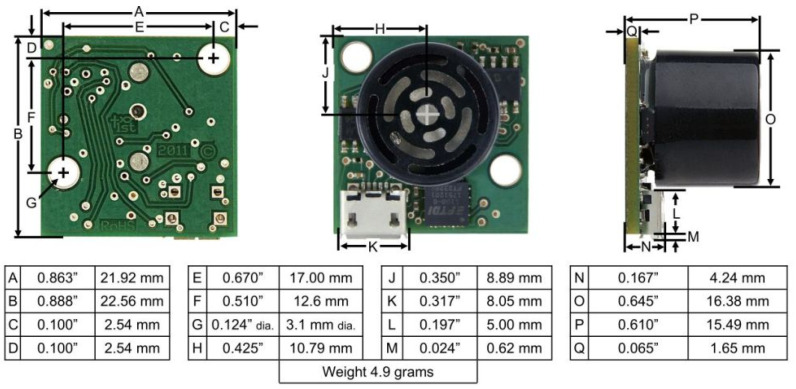
Rear, front, and side representation of the adopted MaxBotix’s MB1403 and MB1433 ultrasonic sensors, together with dimensions and weight.

Illustrative examples of ultrasonic sensors which can be used in IoT-oriented scenarios (and that have been adopted as positioning systems in the remainder of the paper) are the MB1433 [57] and MB1403 [58] sensors produced by *MaxBotix* [59], whose similar external shape is shown in Figure 1.

In detail, they are non-contact ranging sensors using high-frequency ultrasounds in order to detect and estimate objects’ distances within the covered area, in a variety of environments. Their operational principle is the following: they measure the sound’s Time of Flight (ToF) for transmission and back-reflection from nearby obstacles. Then, on the basis of the estimated ToF, the ultrasonic sensor outputs a distance estimate.

Looking at technical specifications, the chosen ultrasonic sensors feature 1 mm resolution, superior rejection of outside noise sources, and internal speed-of-sound temperature compensation. Moreover, despite their ability to detect the presence of objects in the range 1 mm ÷ 5 m, they can practically provide distance estimates in the range 30 cm ÷ 5 m—in fact, objects closer than 30 cm and farther than 5 m are reported as being 30 cm and 5 m far away from the ultrasonic sensor, respectively.

Before providing details on the beam pattern of the chosen ultrasonic sensors, a side note on their internal temperature compensation is of interest. The sound speed in air increases by about 0.6 m/s per degree centigrade [60]. Because of this effect, each MaxBotix device is equipped with an internal temperature sensor performing as follows: if the temperature or humidity changes, the sensor will continue to perform normally over the certified temperature range, compensating for speed-of-sound changes caused by temperature. However, sensors mounted in applications where the environmental temperature gradient is severe may experience a temperature measurement error affecting the sensor’s accuracy. In detail, the recommended operating temperature is in the range 0 ${}^{\circ }C$ ÷ 65 ${}^{\circ }C$, while the recommended dry storage temperature is in the range −40 ${}^{\circ }C$ ÷ 65 ${}^{\circ }C$. Finally, the chosen ultrasonic sensors (i) automatically handle acoustic noise, ignoring a wide plethora of acoustic noise sources; (ii) are not affected by the color, transparency or other visual characteristics of the detected objects, thus being able to be used in dark environments; and (iii) are not highly affected by dust, dirt, or high-moisture environments.

**Figure 2:**
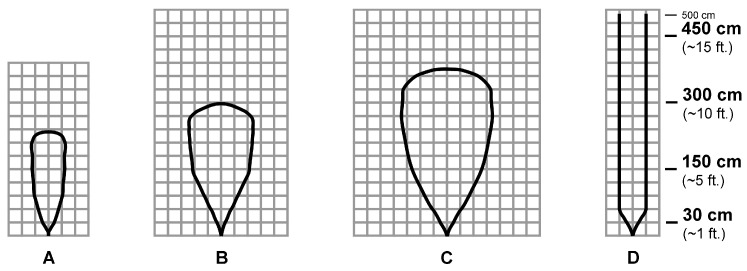
Two-dimensional beam pattern representation of the MaxBotix MB1403 ultrasonic sensor.

**Figure 3:**
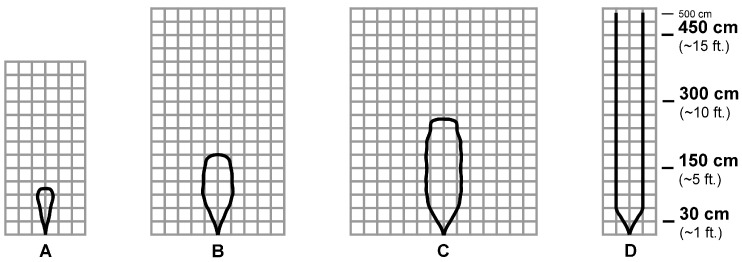
Two-dimensional beam pattern representation of the MaxBotix MB1433 ultrasonic sensor.

The beam pattern of the chosen ultrasonic sensors is described as a two-dimensional irradiation diagram, which represents a projection, over a plane aligned with the sensor’s central axis, of the real detection three-dimensional cone. In general, smaller targets can be detected over a narrower beam angle and at a shorter distance, while larger targets can be detected over a wider beam angle and a longer range. In Figure 2 and Figure 3, the two-dimensional irradiation diagrams of MB1403 and MB1433 sensors are shown, respectively. As a side note, it should be highlighted that (i) beam patterns are in a 1:95 scale for easier comparison on a grid, where a single 1×1 cell corresponds to a 30×30 cm${}^{2}$ area in the real world, and (ii) as detailed in the sensors specifications [61], the detection pattern is for dowels of varying diameters placed in front of each sensor, in both Figure 2 and Figure 3, which are 6.1 mm for case A, 2.54 cm for case B, 8.89 cm for case C, and 27.94 cm for case D.

Analyzing the patterns in Figure 2 and Figure 3, it can be seen how the diagrams in cases A–C differ considerably, while the diagrams in case D are identical. This means that in the case of the detection of objects with a diameter between 6.1 mm and 8.89 cm, the ultrasonic sensors operate differently: in particular, it can be concluded that the beam has a wider and deeper detection range using the MB1403 sensor. In the case of an obstacle with a 27.94 cm diameter up to a distance of 5 m, both sensors behave in the same way. On the basis of this analysis, it can be stated that the MB1403 sensor (i) has a factory-calibrated wide beam width and a high acoustic sensitivity, and (ii) can detect small targets at longer distances: for these reasons, it is mainly suitable for people detection, small target detection, high-sensitivity applications, and obstacle avoidance. On the other hand, the MB1433 sensor (i) has a higher noise-tolerant acoustic sensitivity, (ii) ignores small and medium targets, (iii) detects more targets to long distances, and (iv) has a narrow beam width. Therefore, these features make it suitable especially for large target detection, short-range medium target detection, and applications requiring high noise tolerance.

### 3.2. Sensor Accuracy Characterization

In order to further motivate the utilization of ultrasonic sensors for IoT-oriented environmental perception scenarios, in the following a preliminary accuracy characterization of the chosen sensors is detailed. In particular, three different experimental characterization campaigns were carried out: (i) a *first* evaluation, performed with the ultrasonic sensors fixed at stationary positions; (ii) a *second* campaign, with the ultrasonic sensors aiming forward while approaching perpendicularly a wall at a walking speed; and (iii) a *third* characterization, with the ultrasonic sensors aiming forward while approaching a wall not perpendicularly.

#### 3.2.1. Environmental Characterization Methodology

**Figure 4:**
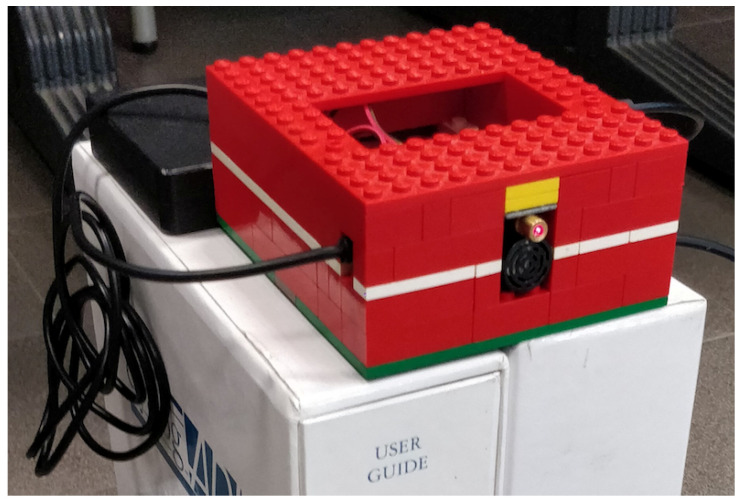
IoT node defined for the experimental characterization campaigns, composed by a Raspberry Pi 3 Model B+, an ultrasonic sensor, and a laser emitter.

The environment where the experimental accuracy characterization has been carried out is a conference room located in a scientific building of the Department of Engineering and Architecture of the University of Parma. In detail, the room was duly emptied in order to have a 25 m${}^{2}$ area with a 2.50 m high wall to be used as a frontal target for the chosen ultrasonic sensors, i.e., a 60 m${}^{3}$ environment. On the practical side, a handmade structure, shown in Figure 4, has been used in order to accommodate the IoT node built for this purpose and composed by (i) a Raspberry Pi 3 Model B+ as processing board, (ii) the ultrasonic sensor to be characterized, and (iii) a laser emitter for wall pointing purposes. In this way, the sensor emission direction is perpendicular with respect to the wall, while the pointing laser can be used to focus the sensor even more precisely than only using the human eye. Finally, it should be noted that, on the basis of the sensors’ specifications, the distance will be estimated starting from the Printed Circuit Board (PCB) surface, and not from the head of the (black) plastic capsule.

#### 3.2.2. Accuracy Characterization with Stationary Ultrasonic Sensors

**Figure 5:**
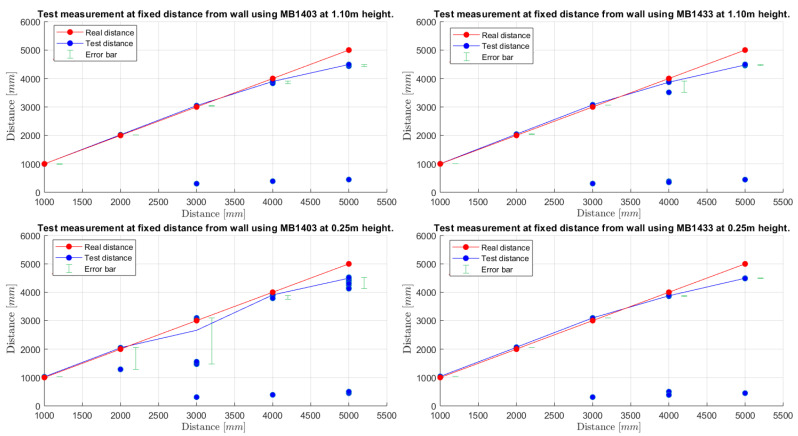
Experimental distance estimates collected with stationary ultrasonic sensors at different distances and heights from the wall.

As introduced in Section 3.2, a first characterization has been carried out with a stationary ultrasonic sensor aiming perpendicularly at a wall at distances from 5 m to 1 m with 1 m step, and at two different heights: 0.25 m and 1.10 m above the ground. Then, for each position (distance, height), an average of 1150 measurements have been collected with a sampling interval of 110 ms, corresponding to a total collection time equal to 1150×110 ms = 126.5 s ≈2 min. The obtained results are shown in Figure 5, where the blue color represents the distance estimate $\hat{d}$ acquired by the IoT node, while the red line corresponds to the real distance *d* from the wall. At each distance the error bar, representing the gap ${\hat{d}}_{\mathrm{best}}-{\hat{d}}_{\mathrm{worst}}$ between the case in which the real distance and the estimated one are closer ($\min\left(d-\hat{d}\right)$) and the case in which the real distance and the estimated one are farthest from each other ($\max\left(d-\hat{d}\right)$), is shown. The isolated blue dots are outliers which are negligible in most cases—note that each dot in Figure 5 corresponds to tens of overlapped measurements, while the outliers correspond to isolated points; they will be discussed in the following. It can be observed that the estimated distance curves have, in all cases, trends similar to the ideal ones, except for the case with the MB1403 ultrasonic sensor placed at a 0.25 m height, which seems to be the case with less-precise results. In particular, it should be noted that the distance estimates have a slight error bar at a real distance of 1 m, 2 m, and 3 m: by placing the sensors at a distance of 1 m and 2 m from the wall, the measured distance is overestimated by 1.64 cm and 4.37 cm on the average, respectively (1.64% and 2.18%, respectively), while at 3 m away it is underestimated by 3.10 cm (1.03%). Moving at 4 m and 5 m from the wall, 11.35 cm and 51.07 cm (2.84% and 10.21%, respectively) of underestimation errors are reached with respect to the real distance, respectively. Therefore, it can be deduced that increasing the distance from the target (the wall) causes an almost linear estimation error increment. This is expected as, by increasing the distance from the target, the ultrasonic pulses are more susceptible to reflections or interference during their paths.

As previously remarked, in Figure 5 it can be observed that at distances equal to 3 m, 4 m, and 5 m, there are clear outliers, i.e., wrong distance estimates (highlighted as strongly isolated blue dots in the bottom part of each graphical plot) between 3000 mm and 5000 mm. These outliers are not due to measurement errors but, rather, to some “misconversions” from the data read by the serial interface of the ultrasonic sensor connected to the IoT node—in fact, the wrong distance estimate ${\hat{d}}_{x}$ at a given step *x* seems to differ from the previous estimate ${\hat{d}}_{x-1}$ and the subsequent estimate ${\hat{d}}_{x+1}$ only in the last digit. In order to avoid this kind of misleading distance estimates, postprocessing techniques—e.g., sliding window filters acting on a few consecutive readings—may be applied.

Therefore, on the basis of the results shown in Figure 5, there seem to be no substantial differences between MB1403 and MB1433 ultrasonic sensors, as they both give similar results when placed at a 1.10 m height. Paying attention to the behavior of the sensors placed at different heights, it can be seen that, at 0.25 m from the floor, the MB1403 sensor is the one with lowest accuracy and highest measurement variance: this could be due to the fact that the beam of this sensor is quite wide and can reach a 2.10 m width (see Figure 2, configuration C). On the other hand, the MB1433 sensor, having a maximum beam width of about 60 cm, does not generate detection errors.

#### 3.2.3. Accuracy Characterization with Moving Ultrasonic Sensors

**Figure 6:**
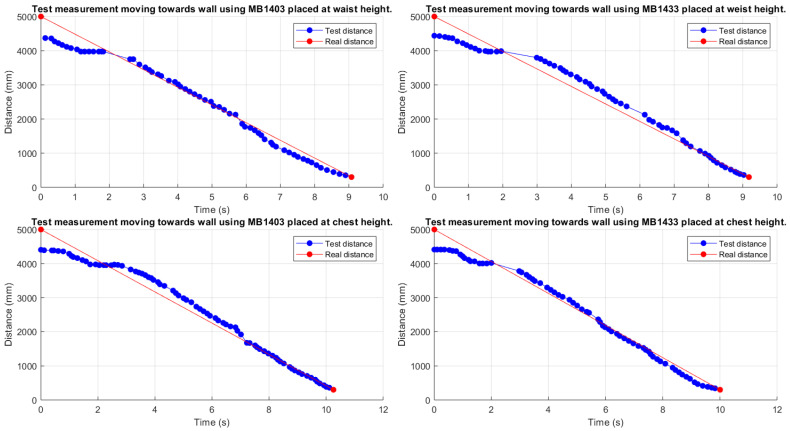
Distance estimates collected with a walk from a 5 m distance from the frontal wall towards the wall itself, wearing the IoT node at waist’s and chest’s height.

Another experimental accuracy characterization, performed using the IoT node described in Section 3.2.1, has been conducted under mobility conditions, as this could represent a more realistic scenario with moving entities (e.g., ultrasonic sensors worn by a person or installed on a vehicle, both moving in their surrounding environments). In this case, distance estimates have been collected starting from a 5 m distance from the frontal wall and walking towards the wall itself, trying to maintain the walking speed as constant as possible. Therefore, in order to make the walk toward the wall as straight as possible, a strip of paper tape has been placed on the ground as a path marker, and the pointing laser has been used to keep the ultrasonic beam pointed at a fixed spot on the wall. Finally, the IoT node has been worn by the test person (i) at chest’s height and (ii) at waist’s height. The experimental results obtained in this mobile configuration are shown in Figure 6, where blue markers represent the distances estimates, and the red line the real distances.

Analyzing the results shown in Figure 6, it can be highlighted that all the cases are affected by an imprecise estimation at the start of the data collection (around 0 s). In fact, the first distances are, on average, around 4.41 m, with a relative error rate equal to 11.88%; this is reasonable, as they are coherent with the results shown in Figure 5 and discussed in Section 3.2.2, in the case of a stationary IoT node at a 5 m distance. Then, while approaching the wall (i.e., for reducing distance between the IoT node and the wall itself), the obtained distance estimate decreases linearly, with smaller and smaller error, i.e., the approaching wall is correctly detected. Moreover, it should be highlighted that, as discussed in Section 3.1, obstacles at a distance shorter than 30 cm are always detected as 30 cm away. Finally, even in this mobile scenario, the obtained results show no substantial behavioral differences from a stationary scenario.

#### 3.2.4. Accuracy Characterization with No-Perpendicular Approaching Direction

**Figure 7:**
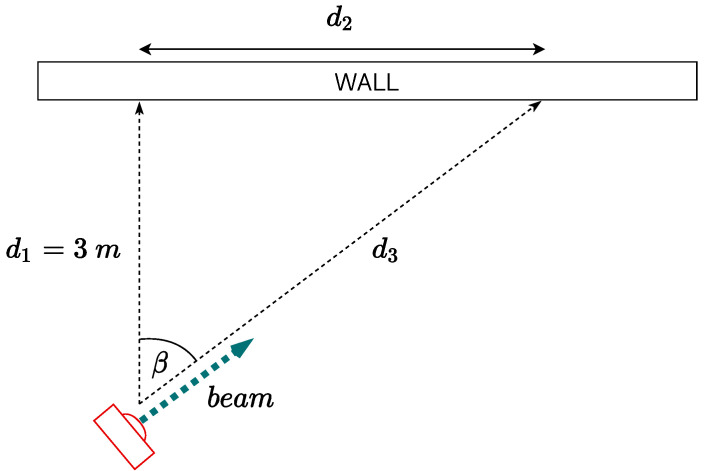
Top view representation of the ultrasonic sensor pointing the frontal wall with a certain incidence angle β.

In addition to the accuracy characterizations discussed in Section 3.2.2 and Section 3.2.3, a further experimental characterization has been performed in order to verify the distance estimates’ accuracy while approaching the wall with a no-perpendicular incidence angle. In order to do this, the IoT node has been fixed at a 3 m distance from the wall, either at a 0.25 m height or at a 1.10 m height, with an incidence angle, with respect to the perpendicular direction towards the wall, in the range $\left[0^{\circ },50^{\circ }\right]$, with a $10^{\circ }$ step. The corresponding geometric scenario, with a generic incidence angle β, is shown in Figure 7.

In Table 2, the obtained distance estimates for various incidence angles are shown. In detail, these results are quite similar for both MB1403 and MB1433 ultrasonic sensors (because of this, in Table 2 no indications on the specific ultrasonic sensor are reported): it can be seen that the distance accuracy degrades for increasing values of the incidence angle, with unreliable estimation for incidence angles above $40^{\circ }$. In fact, increasing the angle beyond $30^{\circ }$, the majority of the resulting distance estimates are equal to 5 m, which is the maximum value detectable by the ultrasonic sensor. This is mostly like due to the fact that by increasing the incidence angle of the acoustic pulses on the wall, these pulses are not reflected perpendicularly and scattered in the environment, making the operational principle of the sensor inapplicable. This implies that detection of side obstacles is a critical aspect for ultrasonic sensors.

## 4. Ultrasonic-Based Environmental Perception

**Figure 8:**
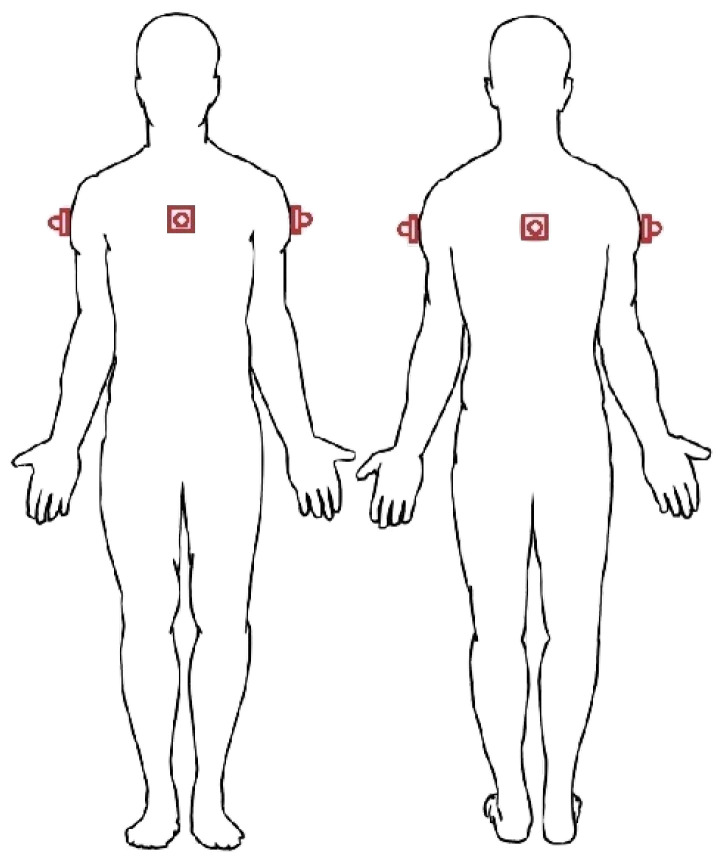
Representation of the ultrasonic sensor disposition for the environmental perception evaluation.

On the basis of the accuracy characterization shown in Section 3.2, we now discuss the reliability of an IoT system, shown in Figure 8 (and similar to the one shown in Section 3.2.1), to perceive the physical topology of the surrounding environment. In particular, we will investigate its sustainability to identify fixed obstacles (even of considerable size), such as walls, doors, gates, or columns. The IoT system is composed by four ultrasonic sensors: (i) a MB1433 sensor placed in the center of the chest (front), (ii) a MB1403 sensor placed in the center of the back (rear), and (iii) two MB1403 sensors attached to the arms (sides). Moreover, this placement is motivated by the interest in perceiving the surrounding environment, rather than detecting obstacles along the path. Therefore, the use of the ultrasonic sensor with the narrowest beam pattern (namely MB1433) in the front does not guarantee accurate obstacle detection, which will be the focus, instead, of Section 5.

The environmental perception evaluation has been performed in two scenarios, walking along straight and non-straight paths, including rotations: (i) the backyard of a house, with three sides corresponding to a wall, a fence, and a garage, and (ii) an indoor small auditorium composed of naves supported by columns.

### 4.1. House Backyard

**Figure 9:**
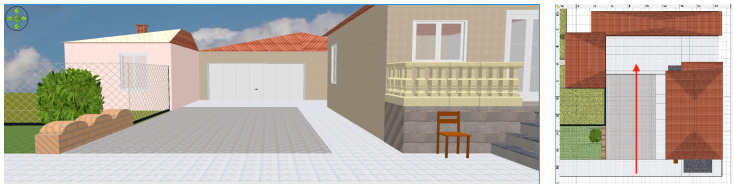
Three-dimensional rendering of the house backyard involved in the environmental perception evaluation. On the right, the walking path is indicated.

With regard to the environmental perception in the house backyard, whose three-dimensional representation is shown in Figure 9, the experimental data were acquired through a walk following a straight path with a 3.50 m width, with a wall on the right and mixed wall–vegetation–fence obstacles on the left. Then, the walk ended 4 m before the front of the garage.

As a side note, in outdoor scenarios (such as this one), it is necessary to pay attention to the weather conditions, as the presence of adverse conditions (e.g., rain or strong wind) can worsen the measurements of ultrasonic sensors, with the audio beam being disturbed during its path.

**Figure 10:**
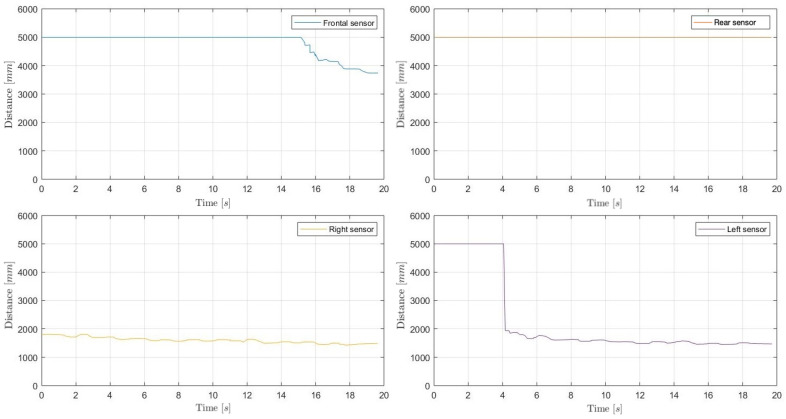
Distance estimates detected with the ultrasonic sensors’ configuration shown in Figure 8, during a walk in the middle of the house backyard.

With reference to the environment shown in Figure 9, the experimental trial has been made walking at an approximate speed of 0.5 m/s in the middle of the backyard, obtaining the results shown in Figure 10.

It can be observed that the forward and backward ultrasonic sensors return a 5 m distance estimate (the maximum), except for the final part of the walk, where the garage is detected by the forward sensor. Moreover, left and right sensors correctly detect the path sides at about 1.70÷1.80 m distances, which is accurate as the track is about 3.50 m width.

### 4.2. Auditorium with Naves Supported by Columns

**Figure 11:**
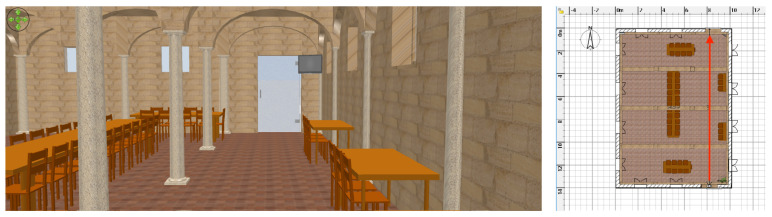
Three-dimensional rendering of the auditorium used for the indoor environmental perception evaluation, located inside the “Santa Elisabetta” Congress Center of the University of Parma. On the right, the walking path is indicated.

An indoor environmental perception evaluation has been carried out at the “Santa Elisabetta” Congress Center of the University of Parma, an old duly-renovated farmhouse provided with three internal naves—each one separated by a series of columns—whose corresponding three-dimensional rendering is shown in Figure 11.

**Figure 12:**
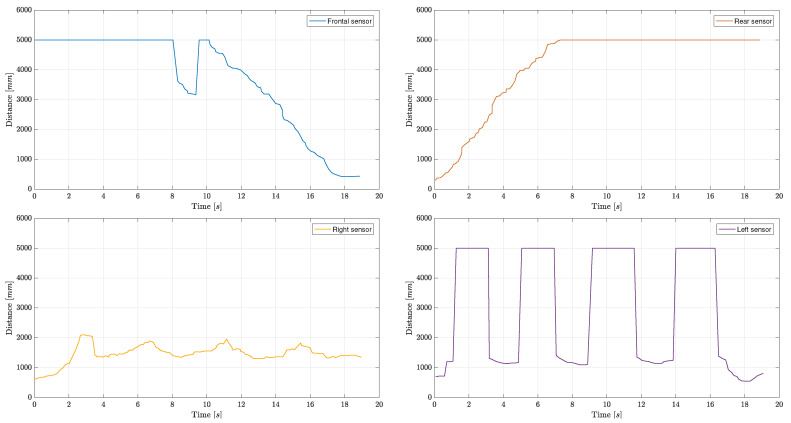
Distance estimates detected with the ultrasonic sensors’ configuration shown in Figure 8, during a walk inside the “Santa Elisabetta” Congress Center of the University of Parma.

In detail, the experimental trial consisted of a straight walk in the middle of the internal aisle, with, on the right, a stone wall with four windows carved into the wall itself and, on the left, three supporting columns along the path. Therefore, the walk started by leaning against the entrance door and ended near the opposite-side door. As can be seen from the results shown in Figure 12, the left-side ultrasonic sensor correctly detected the internal columns during the walk—identified by the periodic distance reduction, down from 5 m to 1.15 m. In particular, the short distance corresponds to the distance between the left shoulder and the nearest column.

Unfortunately, tables and chairs (being 80 cm high) and, in general, small objects, were not detected, as the MB1433 sensor has a narrow beam pattern and, being placed at chest’s height (about 135 cm), small and medium targets cannot be detected (not being included in the beam). On the right side, some peaks can be noticed: they are associated with the windows carved into the wall, placed at 135 cm height on the average. Frontal and rear distance estimation patterns are accurate and denote the displacement from the entrance door (rear sensor) and the approach to the exit door (forward sensor). In the end, these results are quite satisfactory, as the ultrasonic-based perception system can reliably perceive narrow obstacles, such as left-side columns.

## 5. Ultrasonic-Based Obstacle Perception

The environmental perception analysis discussed in Section 4 has highlighted that the considered configuration is not suitable for (especially small) obstacle detection. In the following, we focus on obstacle perception in the outdoor environment shown in Figure 9, considering various configurations of sensors worn by a user: (i) two sensors attached to the waist and pointing in the same direction or with one slightly inclined towards the ground (Section 5.1) and (ii) a pair of sensors at waist’s height, with one pointing forward and one tilted down, and four sensors attached at chest’s height (Section 5.2). In detail, various types of obstacles have been considered: (i) a 18×28 cm size organic waste trash can (low-size obstacle), (ii) a 38×44 cm size glass waste trash can (medium-size obstacle), (iii) a 38×88 cm size large-size obstacle consisting in two trash cans stacked on top each other, and (iv) a 3 cm diameter vertical pole. In turn, each obstacle has been placed in the middle of the house backyard, at a 4 m distance from the garage’s door. For each obstacle configuration, we considered a walk from one side of the backyard towards the obstacle itself, stopping in proximity of the same.

### 5.1. Waist-Attached Adjacent Sensors Pointing Straight Ahead

**Figure 13:**
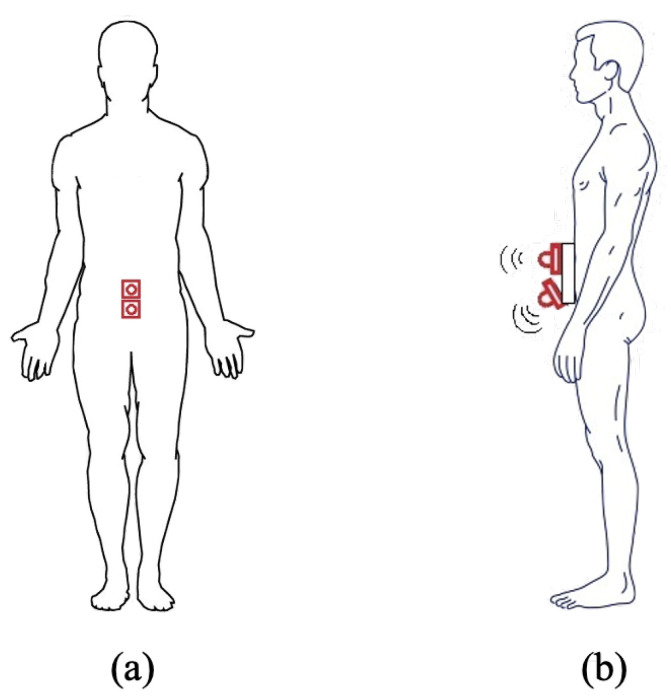
(**a**) Experimental configuration with ultrasonic sensors pointing straight ahead, used for interference evaluation among the sensors themselves. (**b**) Experimental configuration with two ultrasonic sensors, one pointing forward and one tilted down.

The first configuration evaluated for obstacle perception’s purposes is shown in Figure 13a, where two ultrasonic sensors are positioned side-by-side and both point forward. In order to investigate potential interference with each other (*cross-talk*), several trials have been carried out, using a MB1433 sensors as the top one (Sensor1) and a MB1403 sensor as the bottom one (Sensor2), then switching them. Our results show that the adjacency of the ultrasonic sensors is not introducing any cross-talk.

**Figure 14:**
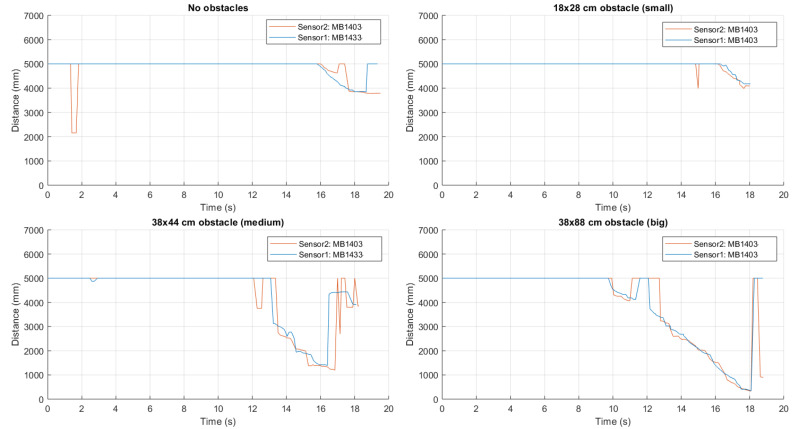
Distance estimates obtained with two adjacent ultrasonic sensors pointing forward.

In Figure 14, we show experimental results in various obstacle configurations: (i) in the unobstructed case, a constant distance estimate of 5 m is observed, followed by a decrease till 4 m (when approaching the garage); (ii) the small-size obstacle is difficult to be identified; (iii) in the case of the medium-size obstacle, it is detected at about 3.70 m and 3.15 m from MB1403 and MB1433 sensors, respectively; and (iv) the large-size obstacle is detected from a distance approximately equal to 4 m. In general, using two adjacent sensors with suitable frontal beam widths can allow to detect a large number of obstacle configurations.

**Figure 15:**
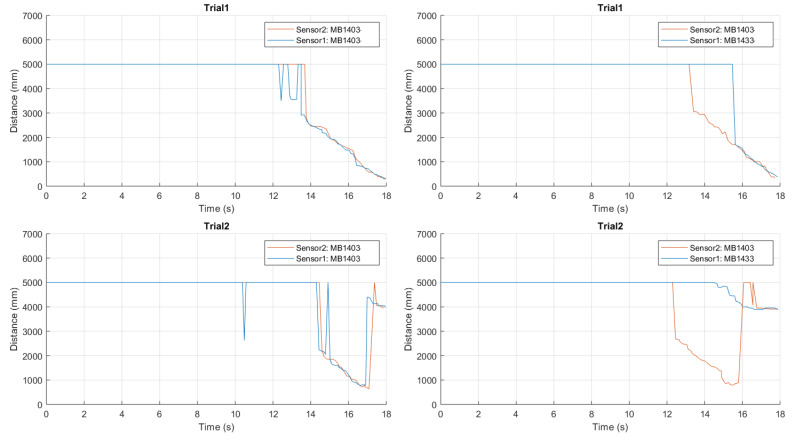
Experimental distance estimates collected with the configuration composed by two adjacent ultrasonic sensors pointing forward, and in presence of a vertical stake-shaped obstacle.

Another evaluation has been performed aiming at identifying a 3 cm diameter vertical pole—such a scenario may be meaningful for urban contexts, during a walk in a street, when users distracted by their phone may risk to bump into a light pole or a road sign, as well as inside cultural buildings, with security barriers or similar. In this context, two situations have been evaluated, as shown in Figure 15: (i) a walk in the backyard, with the pole located in the middle of the walkway, and (ii) a walk in the backyard, not straight towards the pole, but with the pole staying on the walker’s side.

As can be seen from the results shown in Figure 15, in both cases the pole is almost always correctly identified. The only differences are the following:

- this type of obstacle never disappears from the beam, as it is higher than the height at which the sensors are located;
- the MB1403 sensor detects the pole at a distance of 3 m, while the MB1433 sensor (with a more limited beam pattern) detects the obstacle only at a distance of 1.70 m; and
- in the case of the pole side-located with respect to the approaching direction of the user walking in the backyard, the MB1433 sensor cannot detect the obstacle at all, due to its limited beam pattern’s width, which prevents a very narrow obstacle to be detected.

Finally, a modified version of this configuration has been evaluated, tilting the bottom sensor with different inclinations towards the ground, as shown in Figure 13b, and using the MB1403 ultrasonic sensor only (both in the top and bottom positions), due to wider beam pattern and capability to easily detect obstacles.

**Figure 16:**
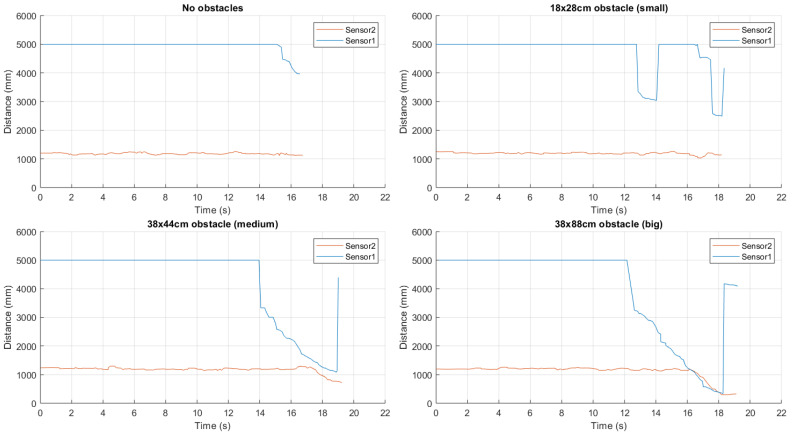
Distances detected with the configuration with two adjacent ultrasonic sensors, the top one pointing forward and the bottom one tilted down with a “medium” inclination.

On the basis of several experimental evaluations performed with the bottom sensor tilted at different inclinations, one needs to find a suitable trade-off to detect heterogeneous obstacles. Our results show that a good trade-off is obtained using a configuration with a “medium” inclination (i.e., on the order of $45^{\circ }$), as shown in Figure 16, where obstacles can be detected from a distance (on average) equal to 1.20 m.

### 5.2. Mixed Chest/Waist-Mounted Ultrasonic Sensors

**Figure 17:**
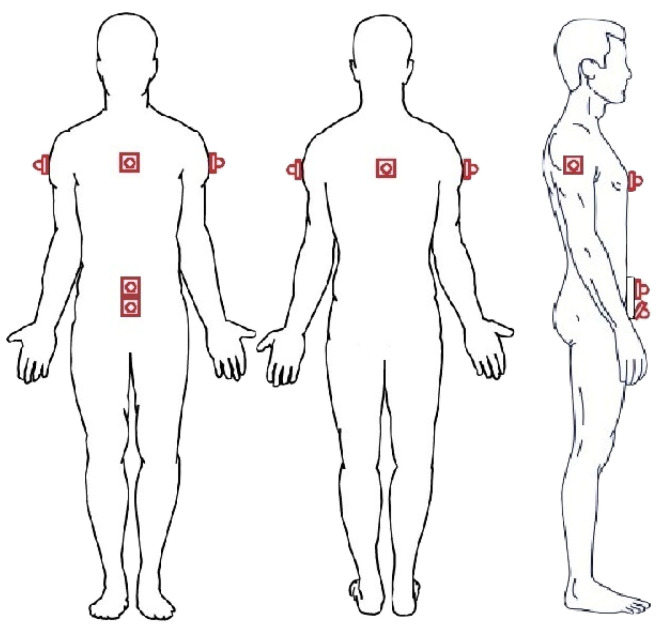
Experimental setup composed by six ultrasonic sensors: one sensor is attached in the middle of the chest, one is in the center of the back, two sensors are attached to the shoulders, and two are fixed to the waist (in turn, one pointing forward and one tilted down towards the floor).

In Figure 17, we show a comprehensive ultrasonic sensor-based IoT system for environmental perception. In detail, the system is composed by two subsets of sensors: (i) four ultrasonic sensors attached at chest’s height (about 130 cm height), with one forward pointing sensor (located in the middle of the chest), one backward pointing sensor (located in the center of the back), and two side pointing sensors (attached to the shoulders); and (ii) two ultrasonic sensors attached to the waist and pointing forward.

**Figure 18:**
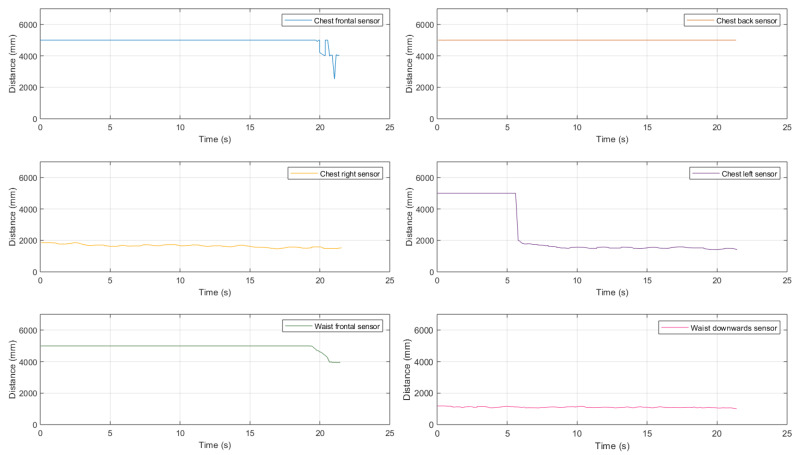
Experimental distance estimates obtained with the configuration shown in Figure 17 during a walk in the backyard of the house, and in the absence of obstacles.

A first trial, whose results are shown in Figure 18, has been carried out in the house backyard with no obstacles.

As can be seen, the distance estimates given by the chest-mounted sensors show results coherent with those shown in the previous scenarios, while the sensors set fixed to the waist is “useless” because there is no need to detect obstacles along the way.

**Figure 19:**
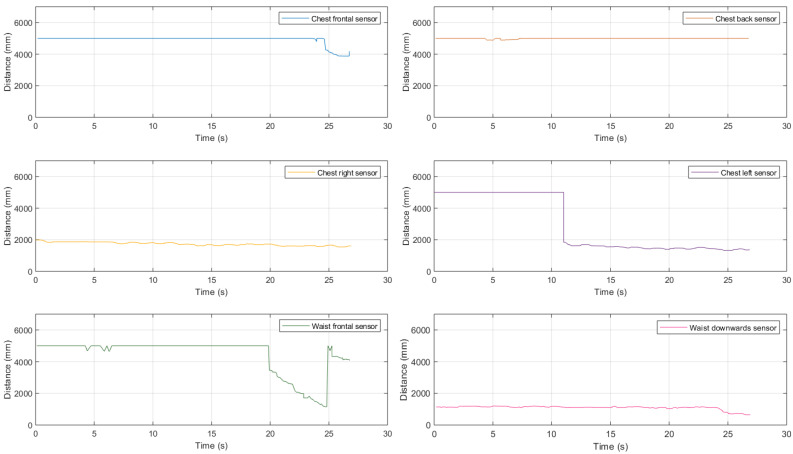
Experimental distance estimates obtained with the configuration shown in Figure 17, in the presence of a 38×44 cm medium-size obstacle along the path.

First, we introduced a medium-size obstacle, obtaining the experimental results shown in Figure 19.

Looking at these results, it can be highlighted the fact that waist-mounted sensors allow to correctly detect the approaching 38×44 cm obstacle, thus avoiding a collision. In particular, the frontal top sensor starts detecting the obstacle at a distance approximately equal to 3.50 m and correctly estimates the distance till it reduces to 1.15 m, when the downward-tilted bottom sensor starts detecting the obstacle itself. Therefore, this represents an interesting validation, highlighting the applicability of the waist-attached sensors to detect an approaching obstacle.

**Figure 20:**
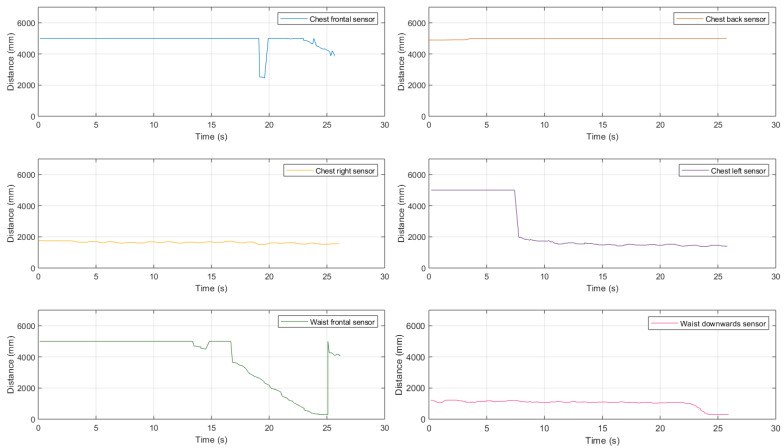
Experimental distance estimates obtained with the configuration shown in Figure 17, in the presence of a 38×88 cm large-size obstacle along the path.

Then, we investigated the performance in the presence of a large-size obstacle, obtaining the results shown in Figure 20.

As for the case with a medium-size obstacle, even in this case the combination of chest- and waist-mounted sensors is helpful.

**Figure 21:**
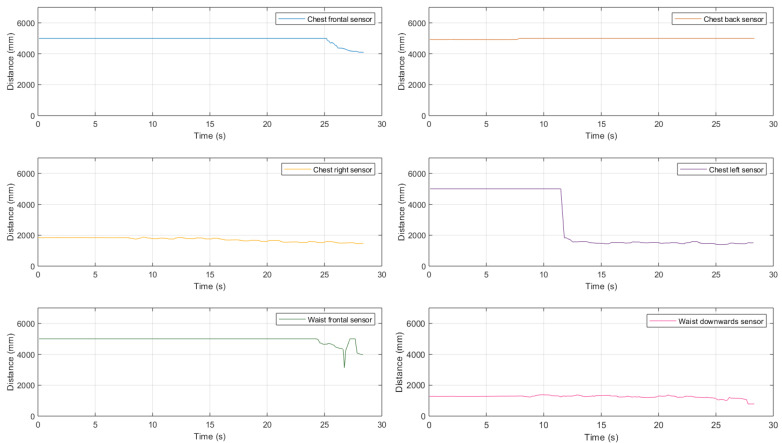
Experimental distance estimates obtained with the configuration shown in Figure 17, in the presence of a 18×28 cm small-size obstacle along the path.

In addition to the experimental tests carried out during a path with medium-size and large-size obstacles, other evaluations were also carried out with a small-size obstacle, obtaining the results shown in Figure 21.

As can be seen from these results, the detection of small-size obstacles (e.g., 18×28 cm) still remains challenging with the proposed ultrasonic sensing system.

**Figure 22:**
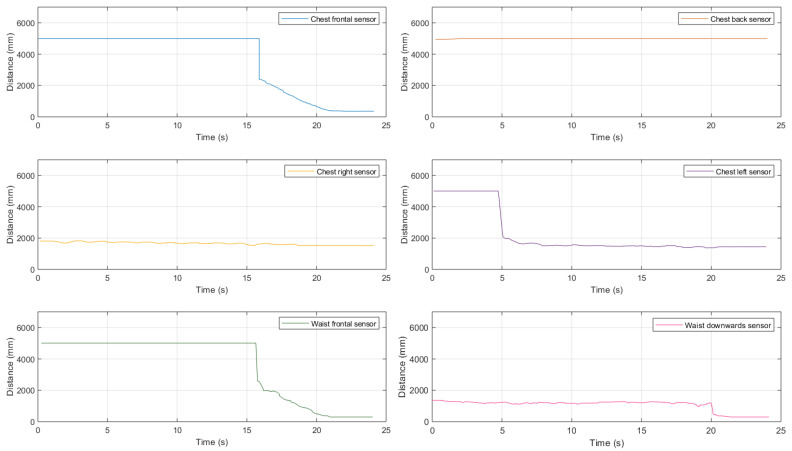
Experimental distance estimates obtained with the configuration shown in Figure 17, in the presence of a stake-shaped obstacle along the path.

Finally, the vertical pole’s detection has been investigated with this configuration, obtaining the results shown in Figure 22.

Even in the presence of an obstacle with such a particular shape, the overall system has an acceptable performance, similar to that of the case of a large-size obstacle. The only difference is that the frontal sensor attached to the chest is sufficient to detect the approaching obstacle—it detects the pole from a distance of about 2.60 m.

On the basis of the obtained experimental performance results, several conclusions can be drawn. *First*, the chest-mounted sensors can be useful to have a rough perception of the surrounding environment. Among the sensors attached to the waist, the frontal one is necessary for the detection of obstacles with both medium (e.g., 38×44 cm) and large (e.g., 38×88 cm) sizes. *Then*, once the obstacles are in the proximity of the frontal sensor, they start being detected by the waist sensor tilted towards the ground, which is also the only one able to detect the proximity of an obstacle of small dimensions (e.g., 18×28 cm). However, the detection in the case of such small objects remains a delicate task, as such obstacles can be detected only at a close range from the walking person.

**Figure 23:**
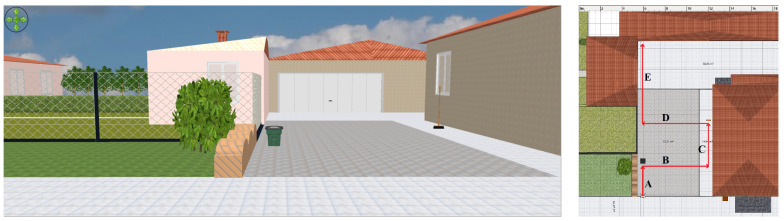
Three-dimensional representation of the backyard, where the red arrows indicate the mixed path followed by the user during the walk.

**Figure 24:**
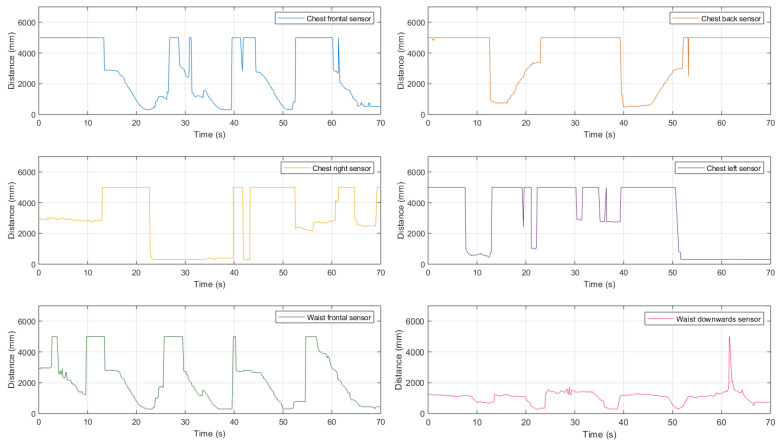
Distance estimates detected with the configuration shown in Figure 17, during a mixed path followed in the house backyard.

Finally, the experimental trial in the house backyard, shown in Figure 23, is considered. This corresponds to a mixed path divided into five phases: (A) the user walks towards a 38×44 cm medium-size organic waste trash can and, in its proximity, he/she deviates to the right with a $90^{\circ }$ direction change; (B) the walk continues towards the house wall and, then, the user turns to the left with a $90^{\circ }$ direction change; (C) the user proceeds along the wall until he/she approaches a vertical pole (namely, a broom with a 3 cm radius), then he/she turns to the left with a $90^{\circ }$ direction change; (D) the walk continues towards the fence, turning to the right at $90^{\circ }$ in its proximity; and (E) he/she walks forward until reaching the garage’s door. The experimental results of the ultrasonic sensor-based system associated with this path are shown in Figure 24.

Looking at the results shown in Figure 24, it seems that this last six ultrasonic sensor-based configuration is the most complete among those discussed in Section 3. Therefore, it represents an effective configuration to perceive the surrounding environment and guarantee the detection of obstacles that may be encountered along the path, regardless of the specific environment and the type of obstacle.

## 6. 5G-Based XR Applications with Ultrasonic Sensor-Based Environmental Perception

Given the experimental evaluations discussed in Section 4 and Section 5, the integration of an IoT environmental perception node based on ultrasonic sensors in a 5G-based XT architecture can be beneficial for a XR end user who needs to move safely in a real environment. On the basis of the results presented in Section 4 and Section 5 (especially regarding obstacles with different sizes) and looking for a safety *add-on* corresponding to an IoT node with highest wearability, a good trade-off is given by the adoption of a single ultrasonic sensor fixed at the user’s waist.

**Figure 25:**
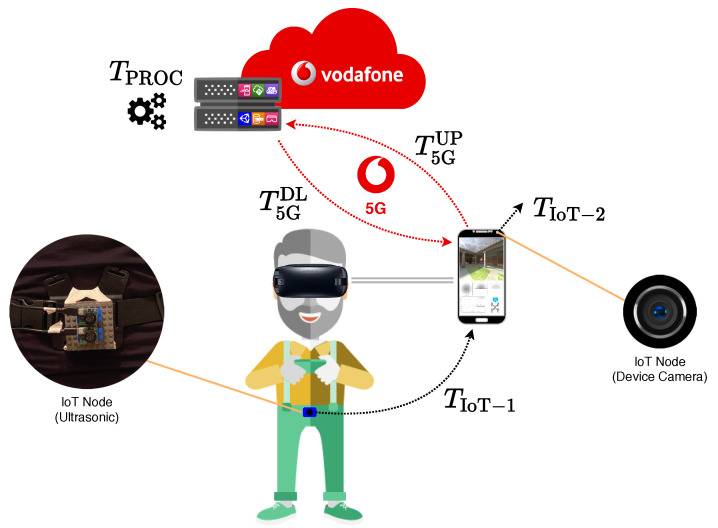
Considered 5G/XR/IoT system architecture.

In Figure 25, the reference architecture, involving IoT devices, a VR visor (which embeds a smartphone), and a high-layer 5G infrastructure, is shown.

In detail, two specific IoT devices are considered: (i) the rear camera of the smartphone embedded in the VR cardboard device—we consider the rear camera of the smartphone as an IoT device, as the proposed approach could be extended to include separate video sensors; and (ii) a waist-mounted ultrasonic sensor system for environmental perception. The second IoT device communicates to the smartphone (e.g., through Bluetooth links), which, in turn, is connected to the 5G network.

According to this system architecture, it is possible to adopt a multi-layer data processing approach, in which data collected from the physical world (through IoT nodes) are first processed “near” the data providers, namely, processed in the same local “ecosystem” collecting the information (e.g., directly inside the IoT node which sensors are connected to), as well as in a first-level aggregation system (e.g., a smartphone) which sensing nodes are sending data to via wireless communication protocols, and, then, sent to higher processing layers for additional (and more computational intensive) processing. To this end, high-layer processing tasks cannot always be needed—depending on the specific application scenario or use case, a first-level data collection, aggregation, and processing could be sufficient to provide an output result to end users, but, in some cases, they may be required because of (i) the need of high-performance infrastructures (e.g., placed in the MEC of the ultra-high-speed cellular network) as well as (ii) the need to consider a large amount of heterogeneous data that are collected from different sources and processed with processing-intensive techniques (e.g., with Artificial Intelligence (AI) algorithms [62]). Currently available cellular networks (4G) have limitations which prevent this approach [40]. With the introduction of 5G ultra-high (in terms of latency and throughput) transmission capabilities, in the architecture shown in Figure 25 the processing is delegated to the network, following an Edge Computing-like approach. As we will see, 5G is crucial owing to the presence of the MEC, which is a major difference with respect to 4G architecture.

### 6.1. XR

Considering industrial trends in gaming and XR-related technologies, as anticipated in Section 1, as well as the need for centralizing content delivery by taking advantage of Edge Computing architectures, 5G allows a paradigm shift when it comes to XR software architectures. The remote rendering and control example of Cloud-based gaming platforms applied to a XR context enables the development of new media and innovative platforms according to a Software-as-a-Service (SaaS) approach. Graphical rendering is then placed on the MEC part of the 5G architecture [63], while the end user’s device becomes a client terminal for streaming the controls of the XR environment to the high-performance computing infrastructure hosted in the 5G Air interface [64,65,66,67]. The XR processing unit on the MEC becomes crucial for the integration and processing of data coming from IoT-based “services”, such as depth or distance measuring sensors (e.g., ultrasonic), IMU sensors, video data (e.g., from cameras), and more. This highlights one more time how future-proof applications will require this kind of technological integration.

As a side note, ultrasound sensors are widely used in robotics for obstacle recognition and tracking with high response rate and accuracy [68,69]. Recent advances in image processing and object detection algorithms have enabled optical camera sensors to be utilized for obstacle detection, especially in the area of autonomous vehicles and self-driven cars [70,71]. However, the response rate is significantly lower and the need for computational power much higher, as one has to capture the image, analyze it, detect the object, decide if an obstacle is recognized, and respond by activating the triggers relevant to each event-based application. The requirement for high computational capacity makes vision-based systems incompatible with the system to be easily worn by end users. Therefore, technologies such as 5G and MEC can be exploited in order to deploy AI-based processing (e.g., image processing through Machine Learning (ML) algorithms) in the Cloud and, with ultra-low-latency, connect the sensor to the Edge and utilize it as a real-time IoT node without the need to have processing power on the end user’s device (e.g., on his/her smartphone). Furthermore, depth sensor and object cloud point algorithms have become widely available [72], enabling easier development and deployment of applications with depth sensing, user 6DoF localization, and object cloud points in a cross-platform manner. The fusion of sensor types proposed in our architecture aims to profit of the best of these worlds: the accuracy and high response and high rate of the ultrasonic sensor, as well as the wide availability and commercialized, undemanding and widely popular usage of camera sensors and depth, position tracking, and image processing algorithms such as ARCore [72]. These benefits could be definitely enhanced with the adoption of modern (commercial) XR systems (e.g., visors) featuring multiple video sensors for detection yet targeting mainstream users. However, in this study we adopted (as will be discussed in Section 7) a simpler VR system, namely, a cardboard containing a commercial smartphone, as it could represent a more viable and portable XR system for an end user, avoiding all the external needs proper of a commercial visor.

### 6.2. 5G

As part of the “5G Trial” promoted by the Italian Ministry of Economic Development (Ministero Italiano dello Sviluppo Economico, MISE) in early 2017, Vodafone implemented in Milan, Italy, a 5G trial network whose main goal was to prove the benefits that the 5G technology could bring to a large number of verticals and use cases with a relevant economic or social impact—areas involved in the trial were Health and Wellness, Security and Surveillance, Smart Energy and Smart City, Mobility and Transport, Manufacturing and Industry, Education and Entertainment, and Digital Divide. We remark that 5G is not only a matter of massive throughput, but it also brings brand new capabilities in terms of ultra-low latency and massive communications. As only the first two characteristics were made available since the beginning—though they will be further improved within the next 3GPP releases—the aforementioned Italian trial mainly concentrated on proving the benefits brought by these features and, according to this perspective, many use cases were designed for.

In order to comply with low latency requirements of these use cases, besides relying on the performance of the NR interface, Vodafone implemented several MEC platforms in different locations within the network. Indeed, thanks to the NFV paradigm, which allows several Core Network Functions (CNFs) to be moved across the network, closer to the 5G User Equipment (UE), the MEC platform also allows to physically bring to the network edge the application functions that, otherwise, would reside on central servers, thus minimizing the latency between client and application. In this way, 5G enables, for the first time, new solutions that would formerly have required a specific fixed connectivity (such as optical fiber) to be performed in mobility over an infrastructured Radio Access Network (RAN). Therefore, it is possible to transfer part of the intelligence from the device to the MEC platform within the 5G network, but preserving the performance in terms of responsiveness. In this way, the computational complexity of the device itself will be reduced, with a simplification of the hardware requirements and a reduction of the costs. This is particularly useful in M2M scenarios, where a very large number of IoT devices can reuse the same intelligence deployed once in the MEC, thus being developed and produced at an affordable cost.

### 6.3. Latency Analysis

Considering the architecture described in this paper and shown in Figure 25, an accurate identification of the latencies involved in the overall data exchange is fundamental in order to guarantee a high UX. To this end, we provide a simple evaluation of uplink (from IoT nodes and VR device to the 5G network) and downlink (from 5G network to the VR device) latencies, taking also into account the processing time in the 5G core network (specifically, in the MEC). The VR system requires gathering data, processing them, and rendering the processed data. Therefore, there is a continuous uplink–downlink data transfer and the overall Round-Trip Time (RTT) has to be sufficiently low to guarantee a real-time perception from the point of view of the VR user. We remark that the 5G network used during the experimental trials in Milan, Italy, and provided by Vodafone, is compliant with the 3GPP 5G R15, operating in the 3.6 GHz transmission band and with Massive Multiple Input-Multiple Output (mMIMO) downlink technology. The QoS condition $T_{\mathrm{RTT}}\leq T_{\mathrm{QoS}}$ could never be satisfied by relying on 4G/4G+ cellular technologies, e.g., with reference to the latency proper of RAN-based architectures, decreasing from tens of ms (LTE) to few ms (5G) [73,74].

According to Figure 25, the smartphone acts as a local collector for the data generated by the considered IoT nodes. Therefore, the uplink latency has two components: (i) the latency required to collect “local” (VR-related and IoT-related) data in the smartphone and (ii) the latency required to transmit these collected data to the 5G network. Concerning the *first latency component*, as the mobile device is integrated in the VR visor, the latency required for VR data extraction is assumed to be negligible; for IoT nodes, the latency is due to the “slowest” IoT node and, therefore, can be written as

(1) $$T_{\mathrm{IoT}}^{\mathrm{UP}}=\max\left\{T_{\mathrm{IoT}-1},\cdots ,T_{\mathrm{IoT}-N}\right\}$$

where $T_{\mathrm{IoT}-i}$ is the latency of the *i*-th IoT device, i∈{1,⋯,N}—in the current scenario, shown in Figure 25, N=2, and recall that we are interpreting the smartphone camera as an IoT sensor, as this set-up could be extended by considering an external IoT video sensor. The *second latency component* is denoted as $T_{5G}^{\mathrm{UP}}$ and depends on the specific smartphone and 5G network characteristics. Finally, the uplink latency can be written as

(2) $$\begin{array}{cc} T_{\mathrm{UP}} & =T_{\mathrm{IoT}}^{\mathrm{UP}}+T_{5G}^{\mathrm{UP}}\\ \; & =\max\left\{T_{\mathrm{IoT}-1},\cdots ,T_{\mathrm{IoT}-N}\right\}+T_{5G}^{\mathrm{UP}}. \end{array}$$

It is clear that if a sensing IoT node has a latency which is too high (with respect to the 5G link latency $T_{5G}^{\mathrm{UP}}$), this is going to degrade the overall uplink latency $T_{\mathrm{UP}}$.

The overall processing time at the MEC is denoted as $T_{\mathrm{PROC}}$ and, depending on processing needs, can assume the following alternative values.

- If VR data processing (corresponding to intermediate VR data processing on the input data to be performed on the end user’s VR equipment—not the final VR data rendering at the MEC) is independent of IoT-based data processing (i.e., IoT data are processed separately from VR data), then
  (3) $$T_{\mathrm{PROC}}=\max\left\{T_{\mathrm{IoT}}^{\mathrm{PROC}},T_{\mathrm{VR}}^{\mathrm{PROC}}\right\}.$$
- if VR data processing is influenced by IoT data processing (i.e., local VR data processing needs to use also IoT input data, in order to generate a final stream to be further processed to produce a final rendering at the end user’s VR equipment), then
  (4) $$T_{\mathrm{PROC}}=T_{\mathrm{IoT}}^{\mathrm{PROC}}+T_{\mathrm{VR}}^{\mathrm{PROC}}.$$

We remark that the *first case*, which is the reference condition considered in this work, corresponds to a scenario where, for example, the IoT environmental data are expedient to provide a warning (e.g., the user is going to hit a wall), but VR processing is independent of it. In the *second case*, instead, VR rendering might be influenced by the IoT sensed data (e.g., there may appear a virtual wall to prevent the user from hitting a real one).

As, in this work, IoT nodes are considered only for the purpose of environmental sensing (not for actuation purposes), the downlink latency corresponds to the transmission of processed data to the VR system. Therefore, the downlink latency $T_{\mathrm{DL}}$ corresponds to the 5G downlink latency, denoted as $T_{5G}^{\mathrm{DL}}$.

In Table 3, we show average values (based on experimental data) for the latency terms involved in uplink, processing, and downlink phases, respectively. More precisely, the experimental data shown in Table 3 have been obtained performing dozens of experimental campaigns in “quasi-static” scenarios (i.e., with a limited degree of mobility of the end user in the environment), with IoT nodes worn by the end user according to the configuration shown in Figure 25. Then, the collected experimental data have been averaged. As highlighted by the results in Section 4 and Section 5, ultrasonic sensors allow rapid estimation of the distance from a frontal obstacle with centimeter-level accuracy. This makes ultrasonic sensors expedient to overcome the limitations generally affecting camera-based environmental detection of VR systems, such as detection of white walls and low obstacles.

At this point, it is possible to evaluate the RTT, denoted as $T_{\mathrm{RTT}}$, which can be written as

$$T_{\mathrm{RTT}}=T_{\mathrm{UP}}+T_{\mathrm{PROC}}+T_{\mathrm{DL}}.$$

As mentioned before, the RTT has to be sufficiently low to guarantee a real-time perception of the VR scenario and an acceptable UX. Denoting as $T_{\mathrm{QoS}}$ the maximum tolerable RTT, a simple constraint is $T_{\mathrm{RTT}}\leq T_{\mathrm{QoS}}$, i.e.,

(5) $$T_{\mathrm{UP}}+T_{\mathrm{PROC}}+T_{\mathrm{DL}}\leq T_{\mathrm{QoS}}$$

which can be rewritten as

(6) $$\max\left\{T_{\mathrm{IoT}-1},\cdots ,T_{\mathrm{IoT}-N}\right\}+T_{5G}^{\mathrm{UP}}+T_{\mathrm{PROC}}+T_{5G}^{\mathrm{DL}}\leq T_{\mathrm{QoS}}.$$

In particular, $T_{\mathrm{QoS}}$ can be interpreted as a proper QoS threshold associated with the required UX.

A state-of-the-art XR-related QoS maximum tolerable latency is $T_{\mathrm{QoS}}≃50$ ms. According to the experimental data shown in Table 3, it holds that the overall RTT $T_{\mathrm{RTT}}≃33$ ms is shorter than $T_{\mathrm{QoS}}$, thus making the proposed approach feasible.

We conclude this latency analysis with a few observations.

- The above latency considerations hold when the IoT system is used for environmental perception, as discussed in Section 4 and Section 5. An alternative scenario could be the one in which processed data have an impact on IoT-oriented actuators. In this case, an additional latency term, associated with the downlink latency needed to reach the actuators, should be considered. The overall downlink latency term could be expressed as $T_{5G}^{\mathrm{DL}}+T_{\mathrm{IoT}}^{\mathrm{ACT}}$, where $T_{\mathrm{IoT}}^{\mathrm{ACT}}$ is the latency to operate an actuator.
- In the considered architecture shown in Figure 25, the VR visor is given by a cardboard embedding a smartphone. An alternative scenario could involve the adoption of a “pure” VR visor, whose data, in turn, should be sent to an intermediate device which would forward them to the MEC through a 5G link. In this case, an additional term $T_{\mathrm{VR}}$ should be considered in the evaluation of both uplink and downlink latencies. Moreover, one could also consider an IoT video sensing node (separate from the smartphone camera).
- In the considered architecture, personal mobility is not expected to be critical with regard to 5G network, as, in the considered quasi-static scenarios, the 5G communication link would not be affected by drastic link quality variations. By contrast, walking mobility has a strong impact on the used IoT sensing technologies, especially ultrasonic sensing: in this case, obstacle distance estimation might be significantly degraded if the end user moves too fast.

## 7. An Application Scenario: Tourism 4.0

Tourism 4.0 [75,76,77] applications may significantly benefit from the use of IoT-enhanced XR systems. In particular, IoT systems can be used to perceive the environment (namely, ultrasonic sensors to perceive obstacles) and/or to navigate the XR user through the real world, while being immersed in a virtual scene. Content-wise and application domain-wise, the area of cultural heritage—in particular Virtual Museums and/or Virtual Cities [63]—is an attractive candidate for the use of the proposed IoT-enhanced XR systems, also because of low-cost cardboards (e.g., as mentioned before, VR headsets holding personal smartphones inside) to maximize their applicability to visitors and audiences. VR-geolocalized contents related to a particular location enable the end user to be fully immersed, with 6DoF free movements in a virtual environment that can provide captivating and emotional experiences associated with the location. Even though IoT sensing nodes, such as ultrasonic sensor-based ones, can then play a significant role in enabling 6DoF motion, they still remain auxiliary protection systems. More precisely, these sensors will not be directly integrated in the 6DoF virtual environment in which the end user is moving, but, rather, will provide auxiliary safety measures required to enable the user to freely walk in his/her surroundings with real-time tracking and obstacle detection (with reference to Section 4 and Section 5, the system can safely detect an approaching obstacle), while fully immersed in the VR environment. This approach allows intensive processing (high quality and resolution rendering) on top of the MEC, streaming to the mobile device, while enabling the feature of inside-out room scanning and 6DoF tracking that only top-level VR headsets include as a feature at the moment.

The development of the platform and application, used also to estimate the latency of the IoT node shown in Section 6.3, has been carried out using Unity v.2018.4.10 and C# as development language [78]. The IoT component of the architecture (namely, the IoT node responsible for obstacle perception and tracking) is handled by a Raspberry Pi 3 (RPi3) hardware board on top of which a Python 3-based library [79] is running to perform different tasks. In detail, the software library is composed by two threads: (i) a *first* software thread, in charge of handling the connection of the ultrasonic sensor to the RPi3 processing board (through a USB cable) and of collecting data, with the library receiving distance estimates via a serial connection at a frequency of about 1000 estimates per second, and (ii) a *second* software thread, hosting a WebSocket-based server [80] and listening on the TCP port 4000, which the VR node, using a WebSocket client, connects to via a Wi-Fi link. Once the WebSocket connection is established, the WebSocket server forwards to the listening clients the distance estimates as soon as they are available from the first software component. Then, the localization component is based on Google’s ARCore and tracks the user’s body in a 6DoF way. The user’s head movements are tracked using the TrinusVR plugin [81], which is also responsible for streaming the head orientation in real-time to the 5G MEC, so that the final image “served” to the end user, denoted as *viewport*, is updated in real-time as well. Using the mobile phone’s camera, the developed algorithm is able to detect depth, as well as to support the IoT component in the detection of obstacles with Points of Interest (POI) recognition in the image frame of the camera. The precision of the tracking algorithm follows the precision of the ARCore library that, for commercial purposes, is able to anchor AR objects in place and track the user around them in real-time, with sufficient accuracy for the successful implementation of AR use cases. We remark that the relative accuracy of user tracking and localization is not the aim of this study, but of future works intended to be conducted by the authors.

**Figure 26:**
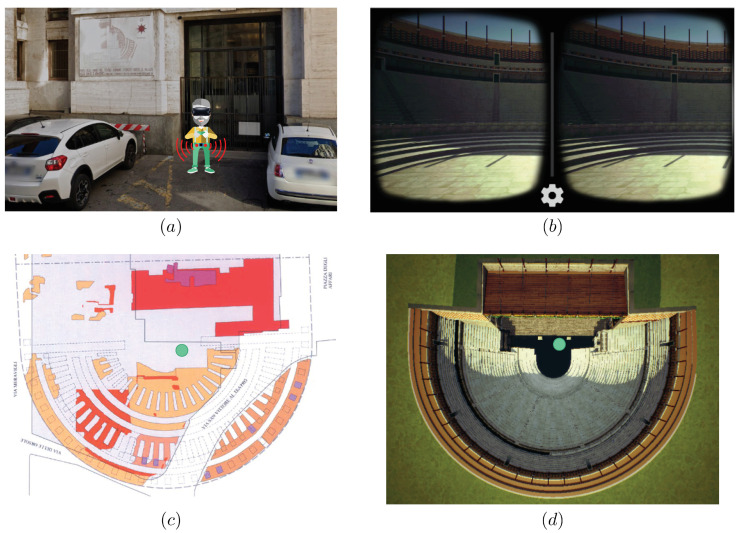
Tourism 4.0 application scenario. (**a**) End user in the real world, with IoT ultrasonic sensor perceiving the environment. (**b**) XR representation processed by the 5G MEC, and returned and showed to the end user inside his/her VR visor. (**c**) Two-dimensional (top view) map representation of the scenario, with a green marker representing the end user position. (**d**) Three-dimensional (top view) representation of the scenario, with a green marker representing the end user position.

Following this approach and the architecture presented in Figure 25, we developed a VR-based IoT-enhanced 5G use case for cultural heritage and touristic attractions content. The overall application scenario is shown in Figure 26. As described in Section 6, a user wearing a VR system is moving in an area of archaeological interest in Milan and an ultrasonic sensor-based IoT system is used to avoid collision with surrounding obstacles (e.g., other tourists or parked cars): this is shown in Figure 26a. Relying on the use of the 5G MEC, the virtual scene is rendered at the network edge and streamed with ultra-low latency through the 5G network to the visor: the view from the visor is shown in Figure 26b. In Figure 26c, the current structure of Milan’s area is overlapped with the old amphitheater which used to be there, which is fully rendered, in the virtual world, in Figure 26d. Finally, in both Figure 26c,d, the end user is located in correspondence to the green dot.

As mentioned before, the system utilizes AR libraries for Simultaneous Localization and Mapping (SLAM) through ARCore through the smartphone’s camera sensor. Initial testing of this set-up highlighted that SLAM technology imposes safety limitations in a VR scenario with free walk without predefined areas. Therefore, the use of a standalone VR headset is not suitable to mobile users. More precisely, SLAM (through ARCore) could not detect white walls and with only a few POIs. Another limitation of ARCore-based SLAM technology is related to the identification of small- and medium-sized objects that could be hazardous obstacles, with the VR user roaming the real environment while immersed in VR. As the instant and precise detection of such obstacles is of paramount importance in this proposed mobile-oriented VR scenario, the IoT ultrasonic sensing node for distance measurement and obstacle presence estimation tasks was crucial to overcome the limitations of the SLAM-based technology. To this end, the smartphone located inside the cardboard becomes a “broker” for IoT nodes and an essential component of the proposed 5G-aided integrated VR-IoT architecture.

## 8. Conclusions and Next Steps

In this paper, we have first discussed the applicability of ultrasonic sensors for environmental perception. Then, we have presented an approach, based on 5G/XR/IoT integration, to allow physical mobility (in the real world) of XR-equipped users with an IoT-based ultrasonic environmental perception system, offloading the overall XR processing to 5G MEC infrastructures. To this end, the combination of IoT and XR information flows, enabled by 5G technology, paves the way to “future-proof” applications, especially mobile applications in the context of the 6DoF concept. The obtained results highlight how QoS and QoE will be key aspects in XR-based systems; latency will thus play a key role. This requires that IoT systems, acting as a support infrastructure for XR-oriented applications, have to be carefully designed to avoid latency limitations. Obviously, 5G plays a key role to guarantee the possibility of delegating to the Edge (namely, to the MEC) intense processing operations. An experimental VR application, denoted as Tourism 4.0 and deployed by Vodafone in Milan, Italy, has been presented.

The results presented and discussed in this paper lead to the following remarks on the proposed 5G/XR/IoT approach.

- As anticipated for the scenarios and use cases discussed in Section 2, even in the context of Tourism 4.0 the adoption of 5G, XR, and IoT paradigms will enhance the experience of end users, making them “live” their surrounding environments, cities, and cultural spaces.
- Latency has to be carefully estimated when a specific scenario needs to be adapted from current cellular communication technologies (namely, LTE cellular networks) to future network technologies and processing paradigms (5G with processing at the MEC). The latency analysis has to take into account all the various system components.
- Each component system to be adopted in the overall architecture has to be preliminary investigated, in order to estimate how its adoption can impact on the overall performance. In particular, proper system configuration has to be considered.

Additional scenarios, which will benefit from the proposed joint 5G/XR/IoT integration and are currently under investigation, are the following.

- Industry 4.0 scenarios, where production machinery’s operational data may be collected through on-field systems (e.g., Programmable Logic Controllers (PLCs)), as well as IoT systems (e.g, ultrasonic sensors, vibration sensors, acoustic sensors, temperature sensors, etc.). Then, workers located inside the production lines can be equipped with ultrasonic sensors and XR visors, used as support devices when a field intervention is needed, in a Maintenance-as-a-Service (MaaS)-oriented way: a technical worker can exploit additional machinery’s contents (e.g., technical documentation, 3D videos, video call with experts working for the machinery’s manufacturer company, etc.), while ultrasonic sensors (worn by the worker) increase his/her safety during the maintenance intervention, warning him/her about the presence of safety barriers that should not be crossed. In this context, 5G technology will allow ultra-fast data transfer and processing offloading from production plants to MEC infrastructures.
- Emergency scenarios involving the intervention of a medical team with its emergency vehicles (e.g., ambulances, medical cars, rescue helicopters, and mountain rescue snowmobiles). When an injured person is reached by rescuers, they may apply IoT health sensors (e.g., breath sensor, continuous glucose monitoring (CGM) and insulin pens, electrocardiogram (ECG), electromyogram (EMG), etc.) on him/her, in order to monitor his/her vital signs, while the doctor can see rescued person’s health parameters through its VR visor. Finally, IoT data can be sent to the hospital emergency room, in which the remote medical staff will receive them together with, possibly, a live video stream kept from the injury scene. On the basis of the received data, the hospital can remotely support rescuers on the accident site, suggesting a first prognosis on the basis of which the injured person may be treated.

## Figures and Tables

**Table 1:** Communication requirements of latency critical IoT applications [41].

Use Case	Latency [ms]	Update Time [ms]	Data Size [bytes]	Communication Range [m]	Mobility [km/h]
Factory automation	0.25 to 10	0.5 to 50	10 to 300	50 to 100	<30
Manufacturing cell	5	50	<16	50 to 100	<30
Machine tools	0.25	0.5	50	50 to 100	<30
Printing machines	1	2	30	50 to 100	<30
Packaging machines	2.5	5	15	50 to 100	<30
Process automation	50 to 100	100 to 5000	40 to 100	100 to 500	<5
Smart grids	3 to 20	10 to 100	80 to 1000	A few m to km	0
Road safety urban	10 to 100	100	<500	500	<100
Road safety highway	10 to 100	100	<500	2000	<500
Urban intersection	<100	1000	1M/car	200	<50
Traffic efficiency	<100	1000	1k	2000	<500
Professional audio	2	0.01 to 0.5	3 to 1000	100	<5

**Table 2:** Real distance ($d_{3}$) and distance estimate (${\hat{d}}_{3}$), obtained varying the incidence angle β in the range $\left[0^{\circ },50^{\circ }\right]$, with a $10^{\circ }$ step. The IoT node is placed at 3 m distance (in the perpendicular direction) from the wall.

β [Degrees]	$d_{2}$ [m]	$d_{3}$ [m]	${\hat{d}}_{3}$ [m]
0	0	3.00	3.00
10	0.53	3.05	3.05
20	1.09	3.19	3.06
30	1.73	3.46	3.06
40	2.52	3.92	–
50	3.58	4.67	–

**Table 3:** Average values (based on experimental data) of the latencies involved in the proposed 5G/XR/IoT architecture.

System	Latency [ms]
Ultrasonic IoT node	$T_{\mathrm{IoT}-1}≃1$
Rear camera sensor	$T_{\mathrm{IoT}-2}≃1$
5G uplink	$T_{5G}^{\mathrm{UP}}≃6$
5G downlink	$T_{5G}^{\mathrm{DL}}≃6$
Data processing	$T_{\mathrm{PROC}}\leq 20$

## Data Availability

Not applicable.

## Abbreviations

The following abbreviations are used in this manuscript:

6DoF	6 Degree-of-Freedom
AR	Augmented Reality
CDN	Content Delivery Network
CGM	Continuous Glucose Monitoring
CNF	Core Network Function
ECG	Electrocardiogram
EMG	Electromyogram
ETSI	European Telecommunications Standards Institute
IEC	Internetional Electrotechnical Commission
IIoT	Industrial IoT
IMU	Inertial Measurement Unit
INS	Inertial Navigation System
IoT	Internet of Things
ITS	Intelligent Transportation System
M2M	Machine-to-Machine
MaaS	Maintenance-as-a-Service
MEC	Multi-access Edge Computing
MEMS	Microelectromechanical Systems
mMIMO	Massive Multiple Input-Multiple Output
ML	Machine Learning
MR	Mixed Reality
NFV	Network Function Virtualization
NR	5G New Radio
PCB	Printed Circuit Board
POI	Points of Interest
PLC	Programmable Logic Controller
QoE	Quality of Experience
QoS	Quality of Service
RAN	Radio Access Network
RTT	Round Trip Time
SaaS	Software-as-a-Service
SDN	Software-Defined Networking
SLAM	Simultaneous Localization and Mapping
ToF	Time of Flight
URLLC	Ultra-Reliable Low-Latency Communication
UE	User Equipment
UX	User eXperience
VR	Virtual Reality
XR	eXtended Reality
